# Development of a novel lysosome-targetable time-gated luminescence probe for ratiometric and luminescence lifetime detection of nitric oxide *in vivo* †
†Electronic supplementary information (ESI) available: Experimental details for the syntheses of **TRP-Tb^3+^** and **TRP-NO**, and supplementary figures. See DOI: 10.1039/c6sc03667h



**DOI:** 10.1039/c6sc03667h

**Published:** 2016-11-23

**Authors:** Zhichao Dai, Lu Tian, Bo Song, Xiangli Liu, Jingli Yuan

**Affiliations:** a State Key Laboratory of Fine Chemicals , School of Chemistry , Dalian University of Technology , Dalian 116024 , P. R. China . Email: bo.song@dlut.edu.cn ; Email: jlyuan@dlut.edu.cn; b School of Chemistry and Chemical Engineering , Linyi University , Linyi 276005 , P. R. China

## Abstract

Rapid, multiplexed, sensitive and specific identification and quantitative detection of nitric oxide (NO) are in great demand in biomedical science. Herein, a novel multifunctional probe based on the intramolecular LRET (luminescence resonance energy transfer) strategy, **TRP-NO**, was designed for the highly sensitive and selective ratiometric and luminescence lifetime detection of lysosomal NO. Before reaction with NO, the emission of the rhodamine moiety in **TRP-NO** is switched off, which prevents the LRET process, so that the probe emits only the long-lived Tb^3+^ luminescence. However, upon reaction with NO, accompanied by the turn-on of rhodamine emission, the LRET from the Tb^3+^-complex moiety to rhodamine moiety occurs, which results in a remarkable increase of the rhodamine emission and decrease of the Tb^3+^ emission. After the reaction, the intensity ratio of the rhodamine emission to the Tb^3+^ emission, *I*_565_/*I*_540_, was found to be 28.8-fold increased, and the dose-dependent enhancement of the *I*_565_/*I*_540_ value showed a good linearity upon the increase of NO concentration. In addition, a dose-dependent luminescence lifetime decrease was distinctly observed between the average luminescence lifetime of the probe and NO concentration, which provides a ∼10-fold contrast window for the detection of NO. These unique properties allowed **TRP-NO** to be conveniently used as a time-gated luminescence probe for the quantitative detection of NO using both luminescence intensity ratio and luminescence lifetime as signals. The applicability of **TRP-NO** for ratiometric time-gated luminescence imaging of NO in living cells was investigated. Meanwhile, dye co-localization studies confirmed a quite precise distribution of **TRP-NO** in lysosomes by confocal microscopy imaging. Furthermore, the practical applicability of **TRP-NO** was demonstrated by the visualization of NO in *Daphnia magna*. All of the results demonstrated that **TRP-NO** could serve as a useful tool for exploiting and elucidating the function of NO at sub-cellular levels with high specificity, accuracy and contrast.

## Abstract 2

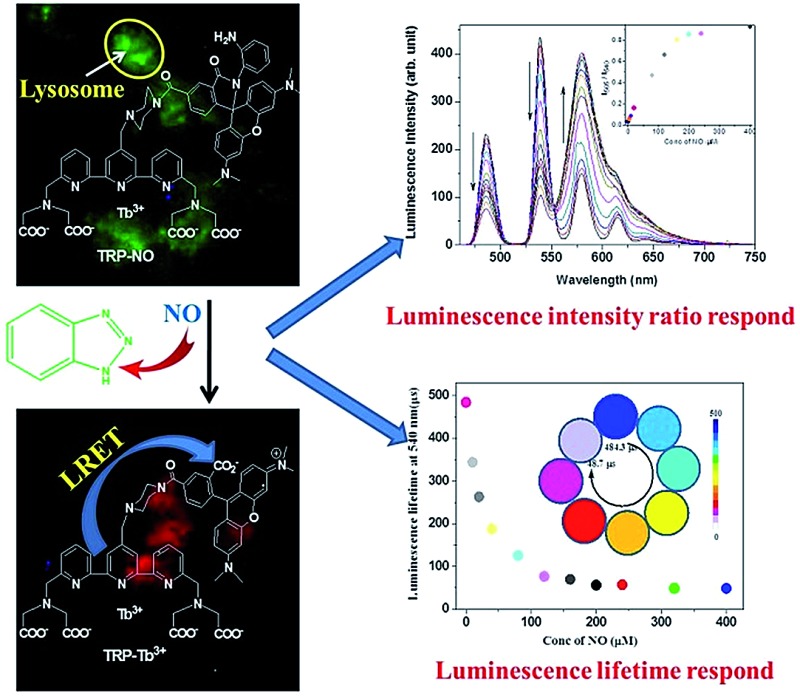
A novel multifunctional probe based on the intramolecular LRET strategy, **TRP-NO**, was designed for ratiometric and luminescence lifetime detection of lysosomal NO.

## Introduction

Nitric oxide (NO), a highly reactive, ubiquitous and uncharged free radical, plays crucial roles in human physiology as an intra- and extracellular messenger molecule.^1,2^ Recent investigations have subverted the view that lysosomal functions are subtly regulated by NO including the degradation of a cell's own components in the catabolic autophagy process to provide the energy and nutrients needed for cell growth through the lysosomal machinery.^3^ Alterations in the level of lysosomal NO were associated with a variety of human diseases, such as Gaucher's disease,^4^ Danon disease,^5^ and lysosomal storage disorders.^6^ Therefore, it is highly desirable to fully understand biological functions of NO, which requires selective and sensitive analytical tools for quantitatively detecting NO in complicated biosystems.

In recent years, the optical bioimaging technique with the use of various molecular fluorescence probes has attracted much attention due to its high sensitivity and selectivity, real-time spatial and temporal resolution ability.^7–9^ A variety of fluorescence probes for NO have been developed, which have remarkably enriched our knowledge on NO homeostasis and its significant roles in biological processes.^10–13^ Unfortunately, few of them were suitable for the quantitative detection of NO in subcellular lysosomes hitherto. One of the challenges is the lack of organelle-specificity, which results in the signal disturbance from cytosol and other organelles. Although two fluorescence probes for the recognition of lysosomal NO were reported quite recently,^14,15^ neither of them could be used for the accurate quantification of NO, which suffers from the interference of photobleaching, microenvironments (such as background autofluorescence derived from the endogenous components of biosamples), and local probe concentration.^16,17^

To overcome the above obstacles, one of the convenient strategies is the employment of ratiometric luminescence probes using lanthanide complexes as luminophores. Lanthanide complex-based luminescence probes exhibit super long-lived luminescence with large Stokes shifts and sharp emission profiles, which enable them to be easily used for time-gated luminescence measurement to eliminate the interference of autofluorescence and scattering lights.^18,19^ Ratiometric fluorescence probes which synchronously record two signals at different wavelengths can get rid of the impact induced by the alteration of excitation intensity, probe concentration, and sample environments.^17^ Hence the combination of ratiometric and time-gated modes is a quite promising approach for the accurate determination of analyte in complicated biosystems. Due to the untunable emission wavelengths of lanthanide complexes, the rational design of lanthanide complex-based ratiometric luminescence probes is still a difficult challenge.^20–22^ On the other hand, the detection of luminescence lifetime (*τ*) instead of intensity has many intrinsic advantages for life-science applications, as the luminescence lifetime is independent of absolute intensity, which is more tolerant of ambient background, electronic noise and varying collection efficiencies.^23^ Therefore, taking luminescence lifetime as the signal for the detection of biological analytes is a potential approach. However, only a few examples for fluorescence lifetime sensing have been reported until now.^24,25^ To the best of our knowledge, a multifunctional luminescence probe that integrates ratiometric and lifetime signals as well as the lysosome-targetable property has not been reported.

**Scheme 1:**
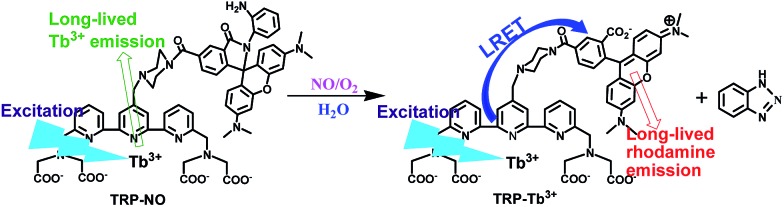
Structure of the probe **TRP-NO** and its luminescence response reaction with NO.

In this work, a unique multifunctional probe, **TRP-NO**, for the sensitive and specific recognition of lysosomal NO with both ratiometric and luminescence lifetime detection modes has been successfully designed and synthesized based on the intramolecular LRET (luminescence resonance energy transfer) mechanism from a luminescent Tb^3+^ complex (LTC) to rhodamine (5-carboxytetramethylrhodamine, CTMR). Before reaction with NO, since the spirolactam derivative of rhodamine moiety in the probe is non-fluorescent, **TRP-NO** emits only the strong Tb^3+^ luminescence. While upon reaction with NO, the spirolactam-ring of the rhodamine moiety is opened, which results in the recovery of the LRET process, meanwhile the time-gated luminescence intensities of LTC and CTMR moieties are remarkably decreased and increased, respectively. The intensity ratio of CTMR/LTC emissions showed a good linearity against the NO concentration. Interestingly, the average luminescence lifetime of **TRP-NO** also showed dose-dependent changes with the variation of the NO concentration, which enabled “luminescence lifetime” to be used as a signal responding to the change of NO concentration. On the basis of these findings, a multifunctional probe equipped with both ratiometric time-gated luminescence intensity and luminescence lifetime responses to NO, **TRP-NO**, was developed. In combination with a true-color time-gated luminescence microscope, **TRP-NO** was successfully used for the imaging of NO in HepG2 cells and *Daphnia magna*. In addition, dye co-localization studies testified to a quite precise distribution of **TRP-NO** in lysosomes by the confocal microscopy imaging. Scheme 1 shows the structure of **TRP-NO** and its luminescence response reaction with NO.

## Results and discussion

### Design, synthesis and characterization of the probe

For obtaining accurately quantitative results in complicated biosystems, the construction of ratiometric time-gated luminescence probes is a promising strategy. Motivated by the widely used FRET mechanism for the design of ratiometric fluorescence probes,^26–28^ we attempted to explore LRET-based ratiometric time-gated luminescence probes by using a lanthanide-complex as the energy donor. In this work, a LRET molecular platform, **TRP-Tb^3+^** (Scheme 1), was initially designed by selecting a luminescent Tb^3+^ complex (LTC) and CTMR as the energy donor and acceptor, which is based on the fact that the emission of LTC has a considerable overlap with the CTMR absorption.^22,29^ Then a NO specifically-reactive moiety, *o*-diaminobenzene, was conjugated to the carboxyl group of the CTMR moiety of **TRP-Tb^3+^**, and a ratiometric time-gated luminescence probe for NO, **TRP-NO**, was obtained. In the absence of NO, the spirolactam derivative of CTMR moiety in **TRP-NO** is on the dark-state, and the probe emits only the Tb^3+^ luminescence. However, after being reacted with NO, the cleavage of the *o*-diaminobenzene leads to the recovery of **TRP-Tb^3+^**, resulting in the restoration of the LRET process from LTC to CTMR. The designed probe was rationally synthesized by marriage of the two fluorophores LTC and CTMR with a rigid linker piperazyl moiety, and was well characterized by NMR, ESI-MS, and CHN elementary analyses.

**Fig. 1:**
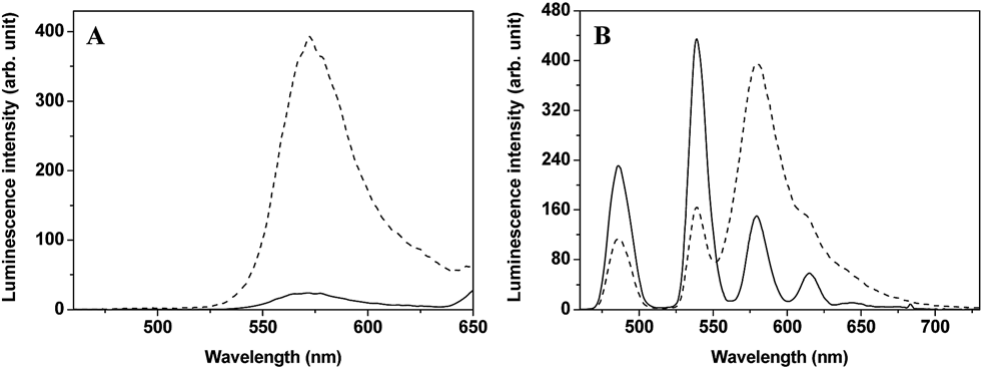
(A) Steady-state emission spectra (*λ*_ex_ = 330 nm) of free CTMR (1.0 μM, solid line) and **TRP-Tb^3+^** (1.0 μM, dashed line). (B) Time-gated emission spectra (*λ*_ex_ = 330 nm) of **TRP-NO** before (solid line) and after (dotted line) reaction with NO (*C*_TRP-NO_ = 15 μM, *C*_NO_ = 0.1 mM, incubation time: 50 min). The solvent was 0.05 M PBS buffer of pH 7.4.

To investigate the luminescence property of **TRP-Tb^3+^**, the steady-state emission spectra of free CTMR and **TRP-Tb^3+^** excited at 330 nm in 0.05 M PBS buffer of pH 7.4 was measured. As shown in Fig. 1A, the Tb^3+^ emission is completely covered by the emission of CTMR moiety in the emission spectrum of **TRP-Tb^3+^**, and the emission intensity of **TRP-Tb^3+^** at 580 nm (the maximum emission wavelength of the CTMR moiety) is 16.4-fold higher than that of free CTMR at the same concentration. By measuring the lifetime of the donor's emission in the absence of acceptor (*τ*_D_, 1.1 ms)^30^ and that of the LRET-induced emission of the acceptor (*τ*_AD_, 0.016 ms), the LRET efficiency (*E*) in **TRP-Tb^3+^** was calculated to be 98.5%, using the equation^31^ of *E* = 1 – (*τ*_AD_/*τ*_D_), which reveals that the energy transfer from LTC to CTMR in the constructed molecular platform, **TRP-Tb^3+^**, is highly efficient. Fig. 1B shows the time-gated emission spectra of **TRP-NO** before and after reaction with NO in 0.05 M PBS buffer of pH 7.4. In the absence of NO, since the spirolactam derivative of CTMR moiety is non-fluorescent, **TRP-NO** emits only the Tb^3+^ luminescence. After being reacted with NO, accompanied by the opening of the spirolactam ring of CTMR moiety, the intramolecular LRET from LTC to CTMR is recovered, so that a dramatic enhancement of rhodamine emission and a remarkable decline of Tb^3+^ emission in the time-gated luminescence emission spectra are observed. Thus it is reasonable to conclude that **TRP-NO** could act as a ratiometric probe for the time-gated luminescence detection of NO with the intensity ratio of rhodamine emission to Tb^3+^ emission as the signal.

### Time-gated luminescence detection of NO in aqueous media using **TRP-NO** as a ratiometric probe

**Fig. 2:**
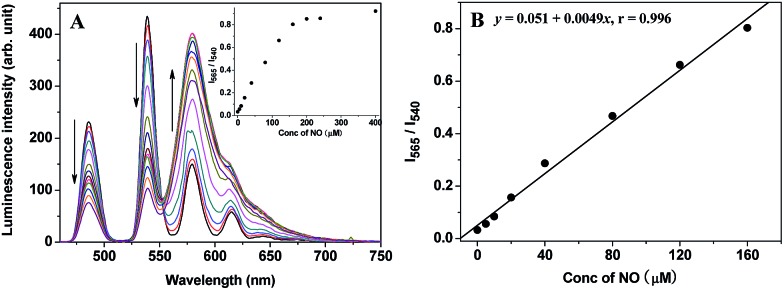
(A) Time-gated emission spectra (*λ*_ex_ = 330 nm) of **TRP-NO** (15 μM) after reaction with different concentrations of NO (0, 5, 10, 20, 40, 80, 120, 160, 200, 240, 320, 400, 640 μM) in 0.05 M PBS buffer of pH 7.4 for 50 min (the inset shows the variation of the *I*_565_/*I*_540_ ratio as a function of NO concentration). (B) Calibration curve for the ratiometric time-gated luminescence detection of NO.

Initially, to evaluate the feasibility of **TRP-NO** as a ratiometric probe for the time-gated luminescence detection of NO in aqueous media, the time-gated emission spectra of **TRP-NO** (15 μM) in the presence of different concentrations of NO (1-hydroxy-2-oxo-3-(3-amino-propyl)-3-methyl-1-triazene, NOC-13, was used as a NO source^32^) were measured in 0.05 M PBS buffer at pH 7.4. As shown in Fig. 2A, upon reaction with different concentrations of NO, the LRET-induced emission of the CTMR moiety at 580 nm was gradually increased, while the Tb^3+^ emissions (a main peak at 540 nm) were apparently decreased. The inset in Fig. 2A shows the correlation between the NO concentration and the intensity ratio of CTMR/Tb^3+^ emissions, *I*_565_/*I*_540_ (the intensity of CTMR emission at 565 nm was adopted to avoid the effect of the Tb^3+^ emission at 581 nm on the CTMR emission at 580 nm (ref. 22 and 29)). With the increase of NO concentration from 5.0 μM to 400 μM, the *I*_565_/*I*_540_ ratio was increased from 0.032 to 0.92, which indicates that a 28.8-fold contrast window can be provided for the detection of NO. Furthermore, the dose-dependent enhancement of the *I*_565_/*I*_540_ ratio showed a good linearity in a wide NO concentration range (Fig. 2B). The detection limit for NO, defined as the concentration corresponding to triple standard deviations of the background signal, was calculated to be 1.8 μM, which suggests that **TRP-NO** could truly be used as a ratiometric probe for the quantitative time-gated luminescence detection of NO. The product of **TRP-NO** reacted with NO was characterized by the mass spectrum detection. As shown in Fig. S5,† the mass spectrum of the product of **TRP-NO** reacted with NO showed three unambiguous signals at *m*/*z* 1190.3 [(M + H)^+^], 1212.3 [(M + Na)^+^] and 1188.3 [(M – H)^–^], which demonstrated the formation of **TRP-Tb^3+^** (MW = 1189.3) as a reaction product, and supported the reaction mechanism of **TRP-NO** with NO shown in Scheme 1.

**Fig. 3:**
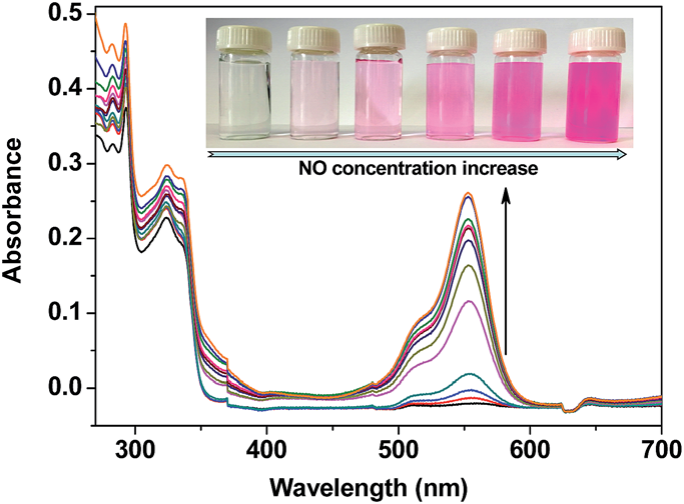
UV-vis absorption spectrum changes of **TRP-NO** (15 μM) after reaction with different concentrations of NO (0, 5, 10, 20, 40, 80, 120, 160, 200, 240, 320, 400 μM) in 0.05 M PBS buffer of pH 7.4 for 50 min (the inserted photographs show colors of the solutions under visible light).

The UV-vis spectrum changes of **TRP-NO** in the presence of different concentrations of NO were further investigated in 0.05 M PBS buffer at pH 7.4. As shown in Fig. 3, after reaction with NO, the absorption of **TRP-NO** at 557 nm was increased significantly. At the same time, the solution's color was remarkably changed from colorless to pink, implying that the spirolactam ring of the CTMR moiety in the probe was opened after the reaction.

### Luminescence detection of NO in aqueous media using **TRP-NO** as a tunable lifetime probe

**Fig. 4:**
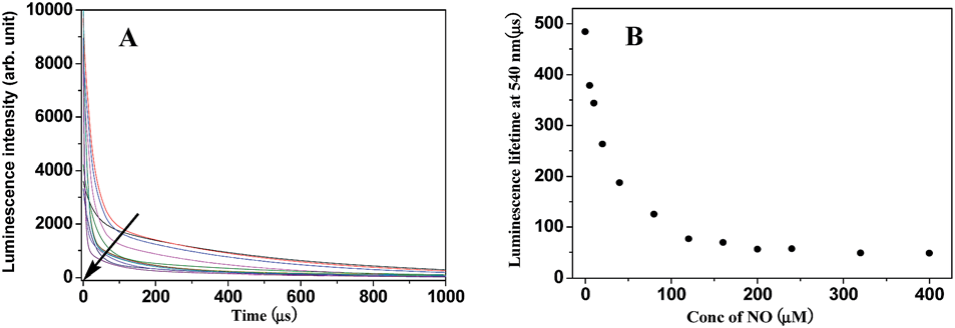
Luminescence intensity decay curves (A) and the average luminescence lifetime changes (B) of **TRP-NO** reacted with different concentrations of NO (0, 5, 10, 20, 40, 80, 120, 160, 200, 240, 320, 400 μM) in 0.05 M PBS buffer of pH 7.4 for 50 min.

Because the intramolecular LRET also dramatically affects the luminescence lifetime of energy donor and acceptor luminophores in the probe, the luminescence decay curves of **TRP-NO** upon reaction with different concentrations of NO were investigated. As shown in Fig. 4A, with the increase in NO concentration, the luminescence decay rate of **TRP-NO** at 540 nm increased gradually, and the average luminescence lifetime (decoded from the luminescence decay curve) of **TRP-NO** decreased gradually from 484.3 μs to 48.7 μs upon reaction with different concentrations of NO (Fig. 4B), which provides a ∼10-fold contrast window for the detection of NO when luminescence lifetime is used as a signal. The results imply that the probe **TRP-NO** could be also used for the luminescence lifetime imaging of NO *in vitro* and *in vivo*. The multiple-signaling feature of **TRP-NO** enables the NO detection to be carried out both with ratiometric time-gated mode and lifetime mode, which affords much convenience for the accurate quantitative detection of NO in complicated biological samples.

**Fig. 5:**
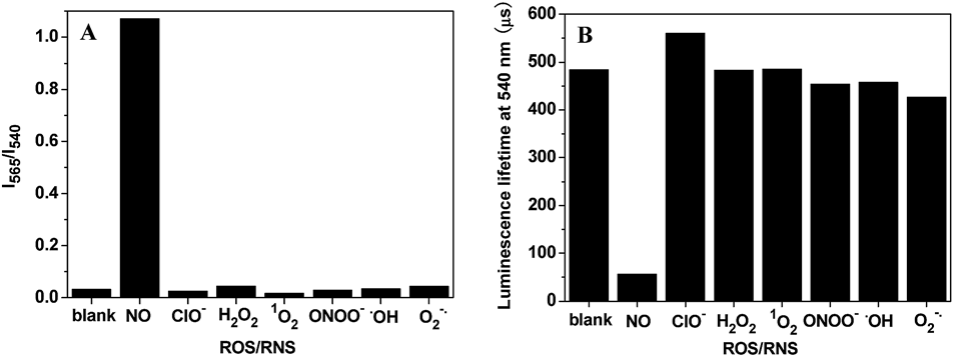
Effects of some ROS/RNS (1.0 mM) on the *I*_565_/*I*_540_ ratio (A) and the average luminescence lifetime (B) of **TRP-NO** (15 μM) in 0.05 M PBS buffer of pH 7.4 for 50 min.

The effects of some reactive oxygen/nitrogen species (ROS/RNS) on the *I*_565_/*I*_540_ ratio and luminescence lifetime of **TRP-NO**, were examined in 0.05 M PBS buffer of pH 7.4. As shown in Fig. 5, both the *I*_565_/*I*_540_ ratio and luminescence lifetime of **TRP-NO** did not show observable responses to the additions of different ROS/RNS including ClO^–^, H_2_O_2_, ^1^O_2_, ONOO^–^, ˙OH and O_2_^–^, while they were remarkably changed after **TRP-NO** was reacted with NO. These results indicate that the luminescence response of **TRP-NO** to NO is highly specific without significant interference from other ROS/RNS both under ratiometric time-gated and lifetime detection modes. In addition, the interference of some metal ions on the *I*_565_/*I*_540_ ratio of **TRP-NO** was further investigated in 0.05 M PBS buffer of pH 7.4 (Fig. S6†). The results revealed that the influence of common metal ions on the *I*_565_/*I*_540_ ratio of **TRP-NO** was negligible.

### Luminescence imaging of NO in living cells using **TRP-NO** as a probe

**Fig. 6:**
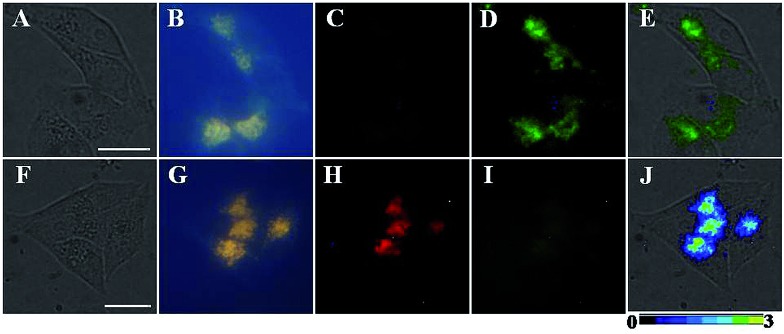
Bright-field (A and F), steady-state (B and G), and time-gated (C and H for rhodamine luminescence, and D and I for Tb^3+^ luminescence) luminescence images of the **TRP-NO**-loaded HepG2 cells before (top) and after (bottom) incubation with NO. Image E is the merged image of bright-field and Tb^3+^ luminescence images of the cells before incubation with NO, and image J is the merged image of bright-field and ratiometric (ratio = *I*_red_/*I*_green_) luminescence images of the cells after incubation with NO. Scale bar: 10 μm.

To evaluate the applicability of **TRP-NO** for imaging NO in living cells, **TRP-NO**-loaded HepG2 cells were prepared by incubating the cells with 200 μM of **TRP-NO** for 4 h at 37 °C in a 5% CO_2_/95% air incubator. After the cells were further incubated with NO (0.5 mM NOC-13 was used) for another 20 min, the cells were subjected to luminescent microscopy imaging measurements. Fig. 6 shows the bright-field, steady-state, time-gated luminescence and ratiometric images of the **TRP-NO**-loaded HepG2 cells before and after incubation with NO (the green filter, 540 ± 25 nm, was used for collecting Tb^3+^ luminescence signals in Fig. 6D and I, and the red filter, >590 nm, was used for collecting rhodamine luminescence signals in Fig. 6C and H). Before incubation with NO, strong, green luminescence signals (Tb^3+^ luminescence) from the **TRP-NO**-loaded cells were observed under time-gated mode (Fig. 6D), while red luminescence signals (rhodamine luminescence) were quite weak (Fig. 6C). However, after the cells were incubated with NO, strong, long-lived red luminescence signals of rhodamine from the cells were observed (Fig. 6H), yet green signals of Tb^3+^ luminescence almost disappeared (Fig. 6I). In addition, compared to the results of steady-state luminescence imaging (Fig. 6B and G), highly specific time-gated luminescence images with unambiguous long-lived green/red luminescence signals (Fig. 6D and H) were obtained, since the autofluorescence has been effectively suppressed by the time-gated mode. The above results indicate that the probe **TRP-NO** could easily enter cells and respond to intracellular NO molecules with the same response behavior as that in the aqueous media. By using the ratio function of ImageJ software, the images collected in red and green filters were evenly divided, and a ratiometric image (ratio = *I*_red_/*I*_green_, scaled in color using ImageJ software) of the **TRP-NO**-loaded HepG2 cells after incubation with NO was obtained (Fig. 6J), which reveals that the reaction products of **TRP-NO** with NO mainly localized in an isolated juxtanuclear area in the cytoplasm of the cells.

**Fig. 7:**
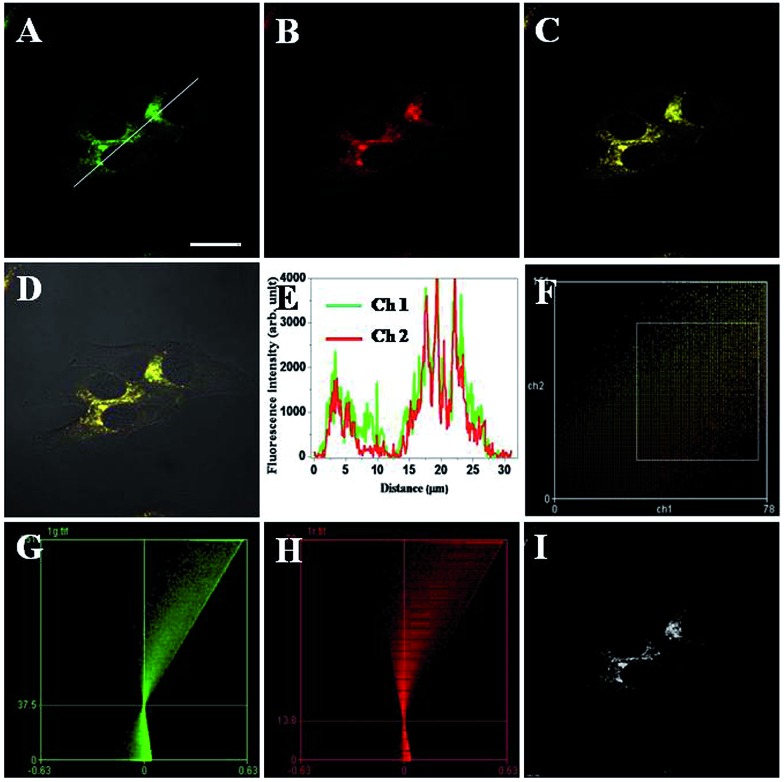
Intracellular co-localization analysis of **TRP-NO** and LysoSensor Green in HepG2 cells. A–C: images of HepG2 cells that were incubated with **TRP-NO**, treated with NOC-13 and further stained with LysoSensor Green (A: channel 1, *λ*_ex_ = 488 nm, *λ*_em_ = 495–545 nm; B: channel 2, *λ*_ex_ = 559 nm, *λ*_em_ = 590–640 nm; C: overlay of A and B). (D) Merged images of C with its DIC. (E) Luminescence intensity profiles of the linear region of interest across HepG2 cells in A. (F) Intensity correlation plot of HepG2 cells co-localized with **TRP-NO** and LysoSensor Green. (G) ICA plots of LysoSensor Green-stained cells. (H) ICA plots of **TRP-NO**-stained cells. (I) PDM image of the cells with positive values in the pixels. Scale bar, 10 μm.

To clarify the nature of intracellular localization of **TRP-NO**, co-localization experiments of **TRP-NO** and two commercially available lysosome-specific fluorescent indicators were performed in HepG2 cells. After the **TRP-NO**-loaded HepG2 cells were treated with 0.2 mM NOC-13 for 20 min and followed by a 10 min-incubation with 1.0 μM LysoSensor Green or 1 h-incubation with 1.0 μM LysoTracker Blue, the cells were imaged on a confocal laser scanning microscope. As shown in Fig. 7 and S7,† the green/blue fluorescence from LysoSensor Green (Fig. 7A) or LysoTracker Blue (Fig. S7A†) and the red fluorescence from NO-reacted **TRP-NO** (Fig. 7B/S7B†) exhibited significant overlap in HepG2 cells (Fig. 7C/S7C†). The intensity profiles of the linear regions of interest across HepG2 cells that co-localized with **TRP-NO** and fluorescent lysosome indicators varied in very close synchrony (Fig. 7E/S7E†). The Pearson's correlation coefficients and the Mander's overlap coefficients are 0.90/0.92 for **TRP-NO**-LysoSensor Green co-loaded HepG2 cells, and 0.93/0.92 for **TRP-NO**-LysoTracker Blue co-loaded HepG2 cells, respectively. All of these coefficients are close to 1, implying that the majority of **TRP-NO** localized within lysosomes of the cells. In addition, the intensity correlation analysis (ICA) was utilized to evaluate the intensity distributions of the co-existing dyes. The intensity of the NO-reacted **TRP-NO** against that of LysoSensor Green or LysoTracker Blue was plotted for each pixel, and highly correlated plots for co-stained HepG2 cells (Fig. 7F/S7F†) were obtained from the dependent staining in Fig. 7C and S7C,† respectively. The ICA plots for the stains of the NO-reacted **TRP-NO** and LysoSensor Green or LysoTracker Blue generate unsymmetrical hourglass-shaped scatterplots that are markedly skewed toward positive values (Fig. 7G and H for **TRP-NO** and LysoSensor Green co-stained cells, and Fig. S7G and S7H† for **TRP-NO** and LysoTracker Blue co-stained cells). Moreover, the intensity correlation quotients (ICQ) for the two stains are 0.367 for the co-existing of **TRP-NO** and LysoSensor Green and 0.321 for the co-existing of **TRP-NO** and LysoTracker Blue, which are close to 0.5, indicating that the stains of **TRP-NO** and the fluorescent lysosome indicators are dependent on each other. Using the results of the differences from the mean (PDM) image with positive values in the pixels (Fig. 7I/S7I†), the lysosomes with high luminescence intensity distributions of **TRP-NO** and the fluorescent lysosome indicators can be easily identified.

The above imaging results of exogenous NO in HepG2 cells revealed the lysosome-targetable feature of **TRP-NO**, which suggests that **TRP-NO** could be a useful probe for the ratiometric time-gated luminescence detection of NO in lysosomes of cells. To confirm this, the feasibility of **TRP-NO** as a probe for imaging the endogenous NO production in RAW 264.7 macrophage cells was investigated. It is known that lipopolysaccharide (LPS) can effectively induce the production of NO in macrophage cells.^33,34^ Thus, after RAW 264.7 cells were incubated with LPS (1.0 μg mL^–1^) for 5 h and followed by a 3 h-incubation with 200 μM **TRP-NO**, the time-gated luminescence images of the cells were recorded using green and red emission filters, respectively. As shown in Fig. S8,† the red time-gated luminescence signals of rhodamine (Fig. S8C†) were obviously observed in the LPS-induced **TRP-NO**-loaded RAW 264.7 cells, while the green signals of Tb^3+^ luminescence (Fig. S8B†) in the cells were rather weak. The ratiometric image (Fig. S8D†) indicates that the ratio signals localized in nonconsecutive vesicles in the juxtanuclear area of cytoplasm, which is quite consistent with the imaging results of exogenous NO in HepG2 cells, demonstrating that **TRP-NO** could truly be used as a ratiometric probe for the time-gated luminescence detection of endogenous NO in lysosomes of living cells.

Because the cytotoxicity characteristic is an important factor to evaluate the practical applicability of a luminescent probe for cell imaging, the influence of **TRP-NO** on cell viability was examined by the MTT assay. As shown in Fig. S9,† the viabilities of HepG2 cells still remained at above 95% after incubation with up to 400 μM of **TRP-NO** for 4 h, which indicates that **TRP-NO** has low cytotoxicity and is biocompatible when it is used as a probe for the imaging of NO in living cells.

### Luminescence imaging of NO in living *Daphnia magna*

*D. magna*, which serve as a keystone species in many ecosystems, are commonly used as sentinel species for contaminant exposure. NO_*x*_ is one type of ubiquitous environmental contaminant, undergoing intracellular conversion, which could be converted to the potent signalling molecule, NO, resulting in the disruption of endocrine-regulated processes.^35^ Therefore, *D. magna* were employed in this work to further confirm the applicability of **TRP-NO** for imaging NO in living systems.

**Fig. 8:**
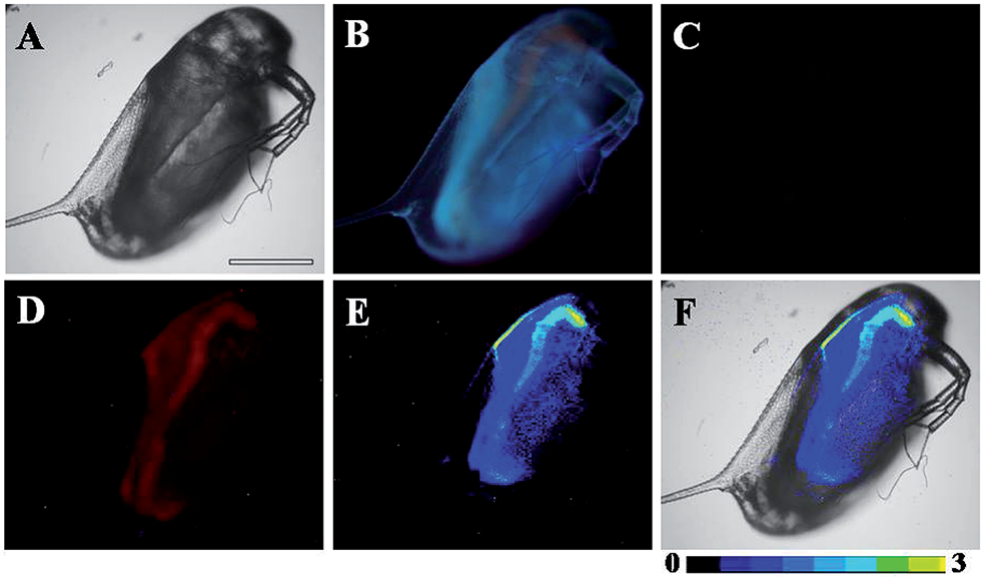
Bright-field (A), steady-state (B) and time-gated (C for Tb^3+^ luminescence, D for rhodamine luminescence) luminescence images of the **TRP-NO**-loaded *D. magna* after incubation with 0.5 mM NOC-13 for 10 min. Image E is ratiometric (ratio = *I*_red_/*I*_green_) luminescence image of the *D. magna*, and image F is the merged image of bright-field and ratiometric images. Scale bar: 200 μm.

As shown in Fig. S10,† before incubation with NO, strong, green luminescence signal (Tb^3+^ luminescence) from the **TRP-NO**-loaded *D. magna* was only observed in the time-gated mode (Fig. S10C†). However, after the **TRP-NO**-loaded *D. magna* were incubated with NOC-13, strong, time-gated red luminescence signals of rhodamine (Fig. 8D) were observed, while green luminescence signals of Tb^3+^ luminescence (Fig. 8C) were quite weak. Compared to the result of steady-state luminescence imaging (Fig. 8B), strong blue autofluorescence from *D. magna* was completely eliminated in the time-gated imaging mode, which allowed the distribution of NO in *D. magna* to be clearly visualized. Furthermore, by using the intensity ratio of rhodamine emission to Tb^3+^ emission, the ratiometric images (Fig. 8E and F) were obtained. The highly specific ratiometric time-gated luminescence images of NO in *D. magna* revealed that the reaction products of **TRP-NO** with NO were mainly located in the intestine of *D. magna* at a higher level than those in abdominal region. These results demonstrated the practical applicability of **TRP-NO** as a ratiometric probe for the time-gated luminescence detection of NO in living bodies, which could be anticipated to be a useful tool for the monitoring of NO_*x*_ contaminants in the ubiquitous environments.

## Conclusions

In this work, based on the intramolecular LRET strategy, a multifunctional probe, **TRP-NO**, has been successfully developed for the time-gated luminescence detection of NO. The probe showed specific luminescence responses to NO, accompanied by the remarkable enhancement of the red rhodamine emission and a significant decrease of the green Tb^3+^ emission, under a single-wavelength excitation, which enabled the *I*_Rh_/*I*_Tb_ ratio of the probe to be used as a signal for the detection of NO. Furthermore, the notable luminescence lifetime declines of **TRP-NO** upon reaction with NO allowed the average luminescence lifetime of the probe to be also used as a signal for the detection of NO. Compared to the previously reported FRET-based or pure lanthanide complex-based luminescent probes,^36,37^ the probe exhibits several distinct merits including large Stokes shifts, long-lived luminescence, good water solubility, and multiple detection modes, which enable the probe to be favourably useful for the ratiometric time-gated luminescence bioimaging and luminescence lifetime bioimaging to monitor the intracellular NO in living samples. The results of ratiometric time-gated luminescence imaging of lysosomal NO in HepG2 cells and *D. magna* demonstrated the practical applicability of the probe. Unfortunately, due to the lack of a suitable luminescence lifetime imaging microscope, a luminescence lifetime imaging experiment is absent. In view of these desirable features, the LRET strategy could be a promising approach for the development of multifunctional probes for the accurate detection of bioactive molecules at sub-cellular levels in bioanalytical and biomedical applications.

## Experimental section

### Materials and physical measurements

Tetraethyl (4′-bromomethyl-2,2′:6′,2′′-terpyridine-6,6′′-diyl) bis(methylenenitrilo) tetrakis(acetate) (compound **6**) was synthesized according to the literature method.^38^ 5-Carboxytetramethylrhodamine (CTMR) was purchased from Zibo Yunhui Bio-technology Co. Ltd. Lipopolysaccharide (LPS) was purchased from Sigma-Aldrich. LysoSensor Green and LysoTracker Blue were purchased from Nanjing KeyGen Biotech Co. Ltd. 1-Hydroxy-2-oxo-3-(3-amino-propyl)-3-methyl-1-triazene (NOC-13) was synthesized according to the literature method.^39^ Tetrahydrofuran (THF) and acetonitrile were used after appropriate distillation and purification. HepG2 and RAW 264.7 cells were obtained from Dalian Medical University. *Daphnia magna* were obtained from Professor Jingwen Chen's group at School of Environmental Science and Technology, Dalian University of Technology. The Krebs–Ringer phosphate buffer (KRP buffer, 114 mM NaCl, 4.6 mM KCl, 2.4 mM MgSO_4_, 1.0 mM CaCl_2_, and 15 mM Na_2_HPO_4_/NaH_2_PO_4_, pH 7.4) was prepared in our laboratory. Deionized and distilled water was used throughout. Unless otherwise stated, all chemical materials were purchased from commercial sources and used without further purification.

^1^H and ^13^C NMR spectra were measured on a Bruker Avance spectrometer (400 MHz for ^1^H NMR and 100 MHz for ^13^C NMR). Mass spectra were measured on a HP1100 LC/MSD MS spectrometer. Elemental analysis was carried out on a Vario-EL analyser. Luminescence lifetimes were measured on an Edinburgh OB920FP fluorescence and phosphorescence lifetime spectrometer. Time-gated luminescence spectra were measured on a Perkin-Elmer LS 50B luminescence spectrometer with the settings of delay time, 0.05 ms; gate time, 0.4 ms; cycle time, 20 ms; excitation slit, 10 nm; and emission slit, 10 nm. All bright-field, steady-state luminescence imaging and time-gated luminescence imaging measurements were carried out on a laboratory-use true color time-gated luminescence microscope.^40^ The confocal fluorescence imaging measurements were carried out on an Olympus FV1000 confocal laser scanning microscope.

### Syntheses of **TRP-Tb^3+^** and **TRP-NO**

The experimental details for the syntheses of **TRP-Tb^3+^** and **TRP-NO** are described in the ESI.†

### Luminescence imaging applications of the probe

Two kinds of cultured cells, human hepatoma cells (HepG2 cells) and murine macrophage cells (RAW 264.7 cells), and laboratory animal *D. magna* were used for the luminescence imaging measurements. The experimental details are described as follows.

#### Time-gated luminescence imaging of exogenous NO in living HepG2 cells

HepG2 cells were cultured on a 35 mm glass-bottom culture dish (*φ* 20 mm) in RPMI-1640 medium, supplemented with 10% fetal bovine serum, 1% penicillin, and 1% streptomycin at 37 °C in a 5% CO_2_/95% air incubator. After removing the culture medium, the cells were incubated with a culture medium containing 200 μM of **TRP-NO** for 4 h. The cells were washed three times with KRP buffer, and further incubated with KRP buffer containing 500 μM of NOC-13 for another 20 min. The cells were washed with KRP buffer, and then subjected to the time-gated luminescence imaging measurements (delay time, 33 μs; gate time, 1.0 ms; lamp pulse width, 80 μs; exposure time, 18 s).

#### Co-localization experiments

After HepG2 cells were incubated with RPMI-1640 culture medium containing 100 μM of **TRP-NO** for 4 h, the cells were washed three times with KRP buffer, and then incubated with KRP buffer containing 200 μM of NOC-13 for another 20 min. The cells were further incubated with LysoSensor Green (1.0 μM) or LysoTracker Blue (1.0 μM) for 10 min or 1 h, respectively. After washing with KRP buffer, the cells were subjected to the confocal fluorescence imaging measurements.

#### Time-gated luminescence imaging of endogenous NO in living RAW 264.7 cells

RAW 264.7 cells were cultured in DMEM medium, supplemented with 10% fetal bovine serum, 1% penicillin, and 1% streptomycin at 37 °C in a 5% CO_2_/95% air incubator. Before loading with **TRP-NO**, the cells were stimulated by LPS (1.0 μg mL^–1^) for 5 h in the culture medium. After washing three times with KRP buffer, the cells were incubated with DMEM medium containing 200 μM of **TRP-NO** for 3 h in the incubator. The cells were washed three times with KRP buffer, and then subjected to the time-gated luminescence imaging measurements (delay time, 33 μs; gate time, 1.0 ms; lamp pulse width, 80 μs; exposure time, 18 s).

#### MTT assay

The *in vitro* cytotoxicity of **TRP-NO** to HepG2 cells was measured by the MTT test using the previously reported method.^41^ HepG2 cells were cultured in a 96-well microtiter plate in RPMI-1640 medium, supplemented with 10% fetal bovine serum, 1% penicillin, and 1% streptomycin, at a density of 5000 cells per well at 37 °C in a 5% CO_2_/95% air incubator. After removing the culture medium, the cells were incubated with the RPMI-1640 culture medium containing different concentrations of **TRP-NO** (0, 50, 100, 200, 300, 400 μM) for 4 h. The cells were washed three times with KRP buffer, and further incubated with KRP buffer containing 833 μg mL^–1^ of 3-(4,5-dimethyl-2-thiazoyl)-2,5-diphenyl-tetrazolium bromide (MTT) for 5 h in the incubator. After removing the supernatants, the cells were dissolved in 100 μL DMSO, and then the absorbance at 490 nm was measured on an Infinite M200 Pro microplate reader.

#### Time-gated luminescence imaging of NO in *D. magna*

The newborn *D. magna* (age < 48 h), cultured in nonchlorinated tap water at 20 °C under cool-white fluorescent light with a 14 : 10 h light : dark photoperiod, were incubated with nonchlorinated tap water containing 100 μM of **TRP-NO** for 1 h at 25 °C. After washing, the *D. magna* were further incubated with nonchlorinated tap water containing 500 μM of NOC-13 for another 10 min. The *D. magna* were washed three times with non-chlorinated tap water, and then subjected to the time-gated luminescence imaging measurements (delay time, 33 μs; gate time, 1.0 ms; lamp pulse width, 80 μs; and exposure time, 15 s).
