# Supplementary material for: Development of a novel lysosome-targetable time-gated luminescence probe for ratiometric and luminescence lifetime detection of nitric oxide in vivo
Source: Chem Sci. 2016 Nov 23;8(3):1969–76. doi: 10.1039/c6sc03667h (PMC5384565; doi:10.1039/c6sc03667h)
Supplement: Supplementary file 1 [file SC-008-C6SC03667H-s001.pdf]

Electronic Supplementary Material (ESI) for Chemical Science.
This journal is © The Royal Society of Chemistry 2016

Electronic Supplementary information for

Development of a novel lysosome-targetable time-gated luminescence probe for ratiometric and luminescence lifetime detection of nitric oxide in vivo

Zhichao Dai,[a,b] Lu Tian,[a,b] Bo Song,*[a] Xiangli Liu,[a] Jingli Yuan*[a]

[a]State Key Laboratory of Fine Chemicals, School of Chemistry, Dalian University of Technology, Dalian 116024, China.

[b]School of Chemistry and Chemical Engineering, Linyi University, Linyi 276005, P. R. China.

*Corresponding authors.

Tel./Fax: +86-411-84986042;

E-mail: bo.song@dlut.edu.cn (B. Song); jlyuan@dlut.edu.cn (J. Yuan).

1. Syntheses of the Ligands (TRP and TR-NO) and Their Tb^{3+} Complexes (TRP-Tb^{3+} and TRP-NO)

The reaction pathway for the synthesis of the ligands TRP and TR-NO is shown in Scheme S1.

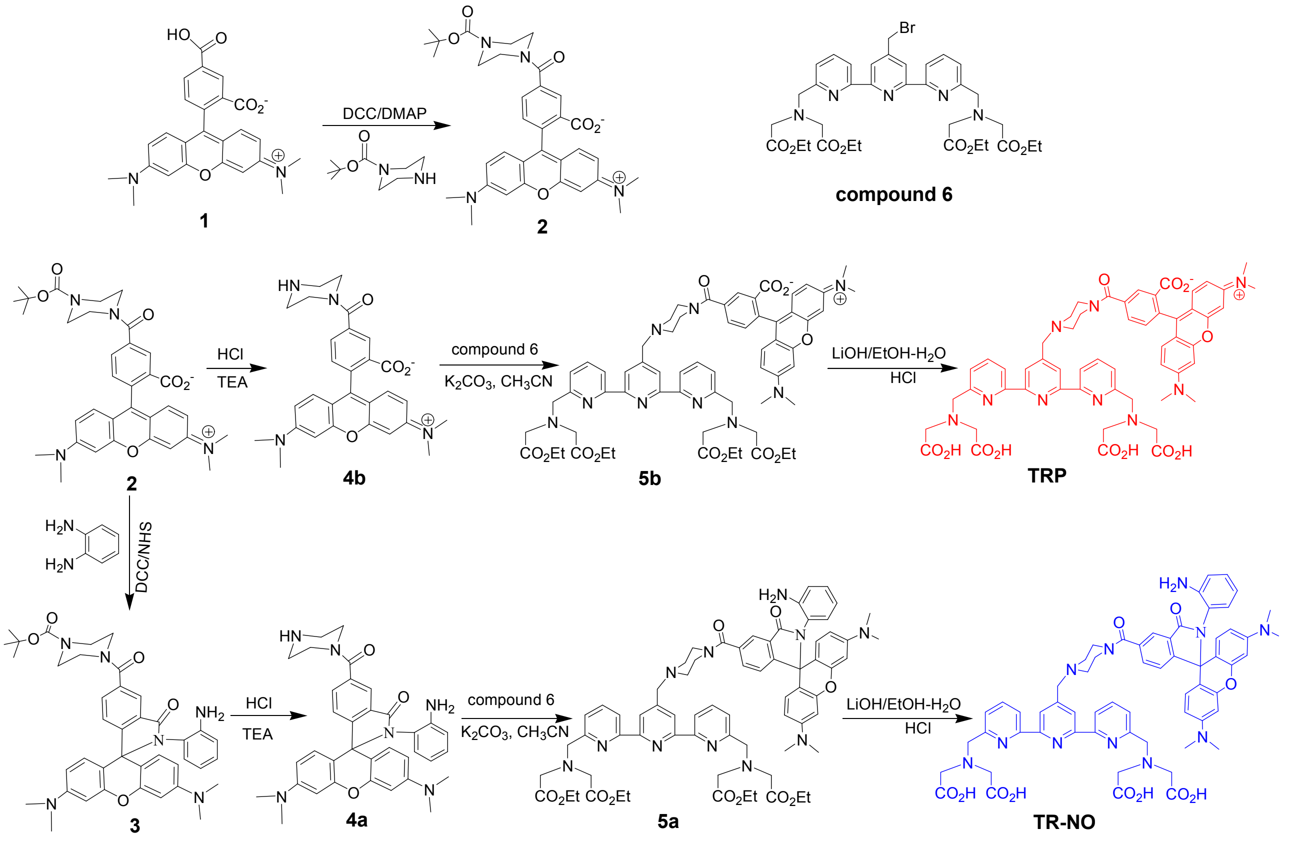

Scheme S1. Reaction pathway for the synthesis of TRP and TR-NO.

The experimental details are as follows.

Synthesis of Compound 2. To a solution of compound **1** (200 mg, 0.47 mmol) in 30 mL of anhydrous CH_3CN, N,N'-dicyclohexylcarbodiinide (DCC, 100 mg, 0.47 mmol) and dimethylaminopyridine (DMAP, 4.2 mg, 0.028 mmol) were added. The solution was stirred for 3 h at room temperature and then mono-*t*-Boc-piperazine (154 mg 0.47 mmol) in 10 mL of anhydrous CH_3CN was added. After the mixture was stirred for another 3 h, the solution was filtered and the filtrate was evaporated. The residue was purified by silica gel column using acetonitrile-water (10:1, v/v) as the eluent. Compound **2** was obtained as a red solid (160 mg, 56.8% yield). ^{1}H NMR (400

MHz, $CDCl_3$): δ = 1.49 (s, 9H), 3.00 (s, 12H), 3.49-3.79 (m, 8H), 6.40 (d, *J* = 8.0 Hz, 2H), 6.49 (d, *J* = 4.0 Hz, 2H), 6.61 (d, *J* = 12 Hz, 2H), 7.23 (d, *J* = 8.0 Hz, 1H), 7.68 (d, *J* = 8.0 Hz, 1H), 8.00 (s, 1H). ESI-MS (m/z): 599.2 $[M+H]^+$.

Synthesis of Compound 3. A mixture of compound **2** (160 mg, 0.27 mmol), N-hydroxysuccinimide (NHS, 115 mg, 0.81 mmol) and DCC (167 mg, 0.81 mmol) in dry CH_2Cl_2 (20 mL) was stirred for 3 h at room temperature. After the mixture was filtered, the filtrate was evaporated, then the residue was re-dissolved in 10 mL of dry CH_2Cl_2 containing 150 μL of triethylamine (TEA). After the *o*-diaminobenzene (59 mg, 0.54 mmol) was added, the solution was stirred for 2 h at room temperature. The solution was evaporated, and the crude product was purified by silica column chromatography, using CH_3CN:H_2O (100:1,v/v) as the eluent. Compound **3** was obtained as a white solid (104 mg, 56.0% yield). ^1H NMR (400 MHz, $CDCl_3$): δ = 1.49 (s, 9H), 2.95 (s, 12H), 3.40-3.60 (m, 8H), 6.08 (d, *J* = 8.0 Hz, 1H), 6.31 (s, 2H), 6.37-6.42 (m, 3H), 6.56 (d, J = 4.0 Hz, 1H), 6.70 (d, J = 8 Hz, 2H), 6.93-6.97 (m, 1H), 7.28, (s, 1H), 7.64 (d, J = 4.0 Hz, 1H), 8.02 (s, 1H). ESI-MS (m/z): 689.4 $[M + H]^+$.

*General Procedure for the Syntheses of Compounds **4a/b***. Compound **2** or compound **3** (0.19 mmol) was dissolved in 10 mL of CH_2Cl_2, then concentrated HCl (120 μL) was added. After stirring for 50 min, the solvent was removed, and the residue was re-dissolved in 5.0 mL of CH_2Cl_2 containing 500 μL of triethylamine (TEA). After stirring for another 15 min, the solution was evaporated, and the crude product was purified by silica column chromatography (using CH_3CN-H_2O (8:1~5:1, v/v) as the eluent) to give compounds **4**.

Compound **4a** was obtained as a white solid (85 mg, 76.0% yield). ^1H NMR (400 MHz, $CDCl_3$): δ = 2.95 (s, 12H), 3.47-3.84 (m, 8H), 6.06 (d, *J* = 8.0 Hz, 1H), 6.30 (s, 2H), 6.36-6.44 (m, 3H), 6.58 (d, *J* = 8.0 Hz, 1H), 6.68 (d, *J* = 8.0 Hz, 2H), 6.94-6.98 (m, 1H), 7.28 (s, 1H), 7.65 (d, *J* = 8.0 Hz,

1H), 8.00 (s, 1H). ESI-MS (m/z): 589.3 $[M + H]^+$.

Compound **4b** was obtained as a red solid (69 mg, 72.9% yield). ^{1}H NMR (400 MHz, D_2O): δ = 3.00 (s, 12H), 3.56-3.84 (m, 8H), 6.23 (d, J = 12.0 Hz, 2H), 6.73-6.76 (m, 2H), 7.10 (d, J = 12.0 Hz, 2H), 7.48 (d, J = 8.0 Hz, 1H), 7.64 (d, J = 8.0 Hz, 1H), 7.83 (d, J = 4.0 Hz, 1H). ESI-MS (m/z): 499.3 $[M]^+$.

*General Procedure for the Syntheses of Compounds **5a/b**.* To a mixture of compound **4** (0.15 mmol) and compound **6** (109 mg, 0.15 mmol) in anhydrous CH_3CN (50 mL) was added anhydrous K_2CO_3 (207 mg, 1.5 mmol). The solution was refluxed overnight, and then cooled to room temperature. After the precipitate was removed by filtration and the filtrate was evaporated, the residue was purified by silica gel column chromatography (using ethyl acetate-petroleum ether (5:1~2:1, v/v) as the eluent) to give compound **5**.

Compound **5a** was obtained as a white oil (97 mg, 52.3% yield). ^{1}H NMR (400 MHz, $CDCl_3$): δ = 1.32 (t, J = 4.0 Hz, 12H), 2.98 (s, 12H), 3.63-3.72 (m, 16H), 4.14-4.19 (m, 14H), 6.07 (d, J = 8.0 Hz, 1H), 6.30-6.41 (m, 4H), 6.68 (d, J = 8.0 Hz, 2H), 6.96 (t, J = 8.0 Hz, 1H), 7.63-7.66 (m, 4H), 7.86 (t, J = 8.0 Hz, 3H), 8.03 (s, 1H), 8.20 (d, J = 4.0 Hz, 1H), 8.43 (s, 2H), 8.50 (d, J = 8.0 Hz, 2H). ESI-MS (m/z): 1258.5 $[M + Na]^+$.

Compound **5b** was obtained as a red oil (69 mg, 40.1% yield). ^{1}H NMR (400 MHz, $CDCl_3$): δ = 1.20 (t, J = 8.0 Hz, 12H), 2.94 (s, 12H), 3.44-3.75 (m, 16H), 4.06-4.12 (m, 14H), 6.39-6.46 (m, 4H), 6.57-6.63 (m, 2H), 7.19 (d, J = 8 Hz, 1H), 7.57 (d, J = 8.0 Hz, 2H), 7.68 (d, J = 8.0 Hz, 1H), 7.87-7.93 (m, 3H),8.36 (s, 2H), 8.44 (d, J = 8.0 Hz, 2H). ESI-MS (m/z): 1146.5 $[M]^+$.

General Procedure for the Syntheses of the Ligands (TRP and TR-NO). To a solution of compound **5** (0.083 mmol) in 13 mL of ethanol was slowly added the solution of LiOH (20 mg, 0.83 mmol) in 1.5 mL H_2O within 2 h with stirring. After the mixture was stirred at room

temperature for 20 h, the solvent was evaporated, and the residue was dissolved in 5 mL of water. The solution was acidized to pH ~3 with HCl (3 M), and the suspension was stirred for another 3 h at room temperature. The precipitate was collected by filtration. After drying, the precipitate was added to 15 mL of dry acetonitrile, and the mixture was refluxed for 20 min. The precipitate was filtered and dried again to afford the relative TR-ligand.

TRP was obtained as a red solid (69 mg, 80.4% yield). ^{1}H NMR (400 MHz, DMSO-d_6): δ = 2.99 (s, 12H), 3.50-3.58 (m, 12H), 3.75-3.82 (m, 4H), 4.11 (s, 6H), 6.58 (s, 4H), 6.66 (d, J = 8.0 Hz, 2H), 7.31 (d, J = 8.0 Hz, 1H), 7.63 (d, J = 8.0 Hz, 2H), 7.77 (d, J = 8.0 Hz, 1H), 7.80 (t, J = 4.0 Hz, 3H), 8.41 (s, 2H), 8.49 (d, J = 8.0 Hz, 2H). ^{13}C NMR (100 MHz, DMSO-d_6): δ = 45.88, 51.62, 53.30, 58.12, 59.71, 95.85, 108.93, 118.15, 119.66, 122.03, 124.43, 128.49, 129.13, 132.85, 135.67, 136.75, 147.94, 151.75, 151.90, 153.22, 153.87, 154.64, 157.66, 163.11, 165.16, 166.39, 166.63, 171.30, 196.73. Elemental analysis calcd (%) for $C_{55}H_{56}N_9O_{12}\cdot 5H_2O$: C 58.71, H 5.91, N 11.20; found (%): C 58.69, H 6.13, N 10.95. ESI-MS (m/z): 1034.4 $[M]^+$.

TR-NO was obtained as light pink powder (70 mg, 75.1% yield). ^{1}H NMR (400 MHz, DMSO-d_6): δ = 2.89 (s, 12H), 3.54-3.57 (m, 12H), 3.73-3.81 (m, 4H), 4.10 (s, 6H), 5.89 (d, J = 4.0 Hz, 1H), 6.24-6.27 (m, 3H), 6.42 (d, J = 8.0 Hz, 2H), 6.53 (d, J = 8.0 Hz, 1H), 6.71 (s, 1H), 6.86 (t, J = 8.0 Hz, 1H), 7.11-7.18 (m, 1H), 7.60-7.64 (m, 3H), 7.83 (s, 1H), 7.97-8.02 (m, 2H), 8.27 (s, 1H), 8.41-8.44 (m, 2H), 8.51 (d, J = 8.0 Hz, 2H). ^{13}C NMR (100 MHz, DMSO-d_6): δ = 54.87, 55.34, 59.69, 59.87, 67.42, 67.52, 98.50, 109.11, 115.97, 116.41, 119.80, 121.03, 121.33, 121.64, 123.67, 124.66, 128.77, 129.32, 132.08, 136.61, 138.28, 138.41, 146.18, 151.57, 153.13, 154.10, 154.78, 154.98, 155.30, 155.49, 159.22, 159.39, 165.30, 168.71, 172.93. Elemental analysis calcd (%) for $C_{61}H_{61}N_{11}O_{11}\cdot 5H_2O$: C 60.34, H 5.89, N 12.69; found (%): C 60.37, H 5.57, N 12.92. ESI-MS (m/z): 1122.7 $[M - H]^-$.

General Procedure for the Syntheses of the Tb^{3+} Complexes (TRP-Tb^{3+} and TRP-NO). Two Tb^{3+} complexes, TRP-Tb^{3+} and TRP-NO, were synthesized by in-situ mixing equivalent molar of the ligand (0.01 mmol) and $TbCl_3{\cdot}6H_2O$ (0.01 mmol) in 5.0 mL of 0.05 M PBS buffer of pH 7.4. After stirring for 30 min at room temperature, the solutions (stock solutions) were stored at room temperature, and suitably diluted with aqueous buffers before use.

2. Supplementary Figures

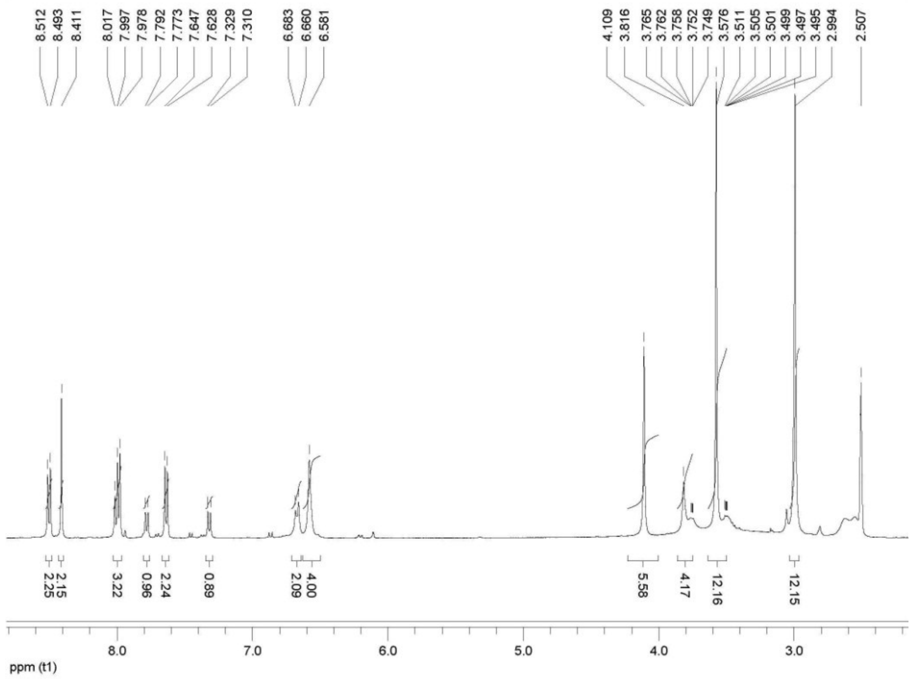

Fig. S1. ^{1}H NMR spectrum of the ligand **TRP** (400 MHz, DMSO-d_6).

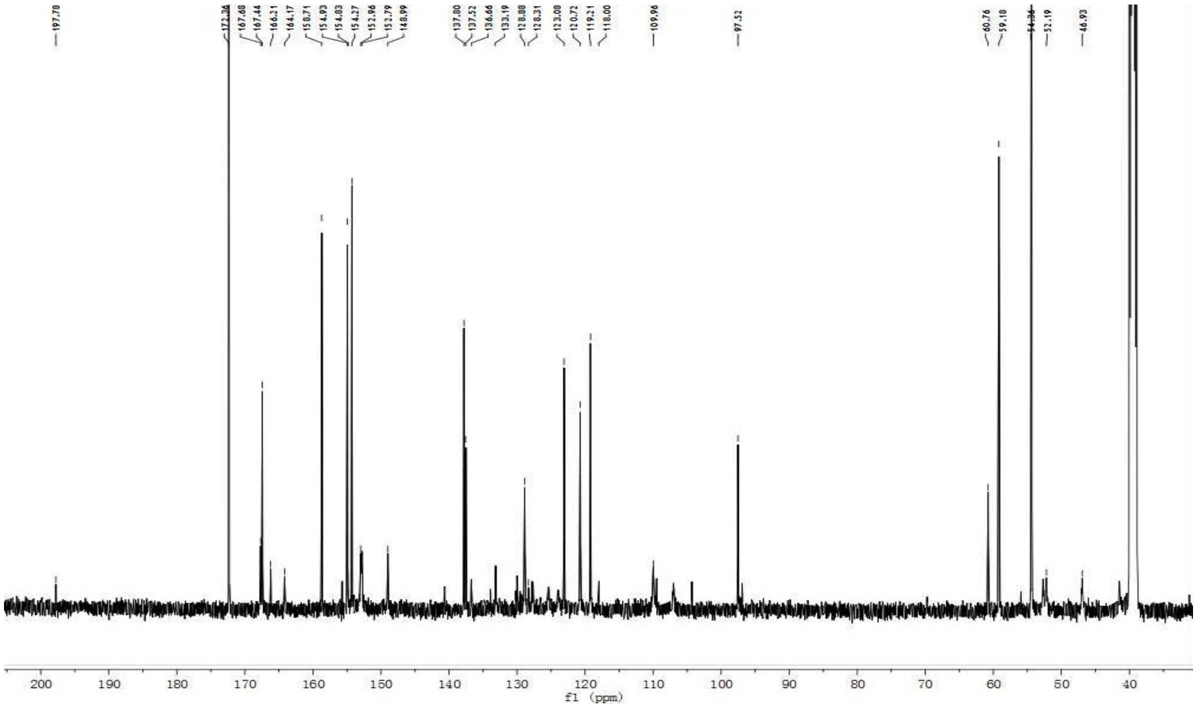

Fig. S2. ^{13}C NMR spectrum of the ligand **TRP** (100 MHz, DMSO-d$_6$).

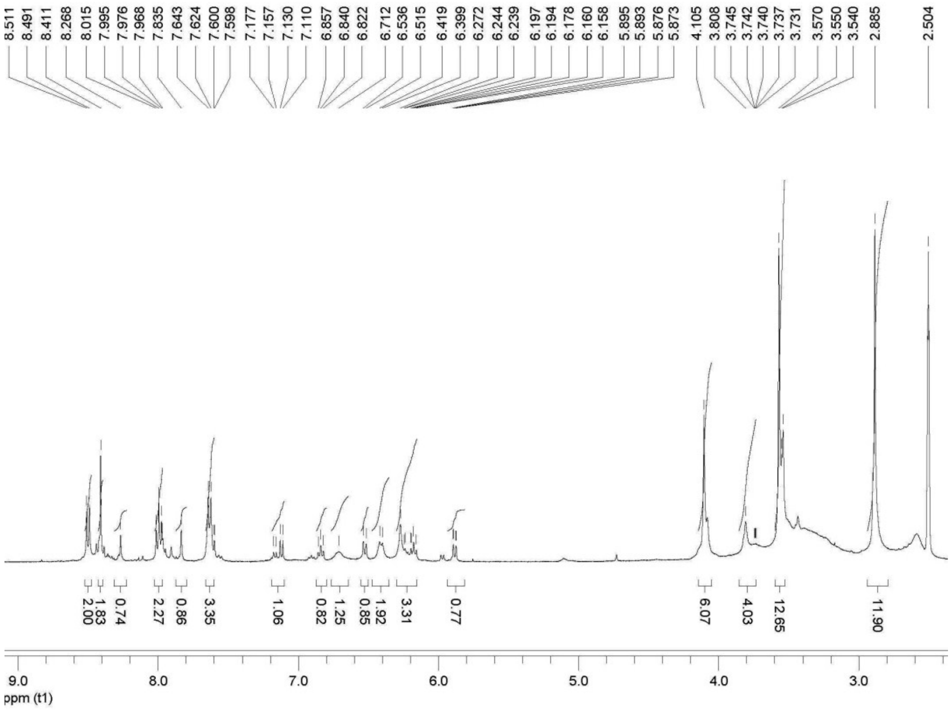

Fig. S3. ^{1}H NMR spectrum of the ligand **TR-NO** (400 MHz, DMSO-d$_6$).

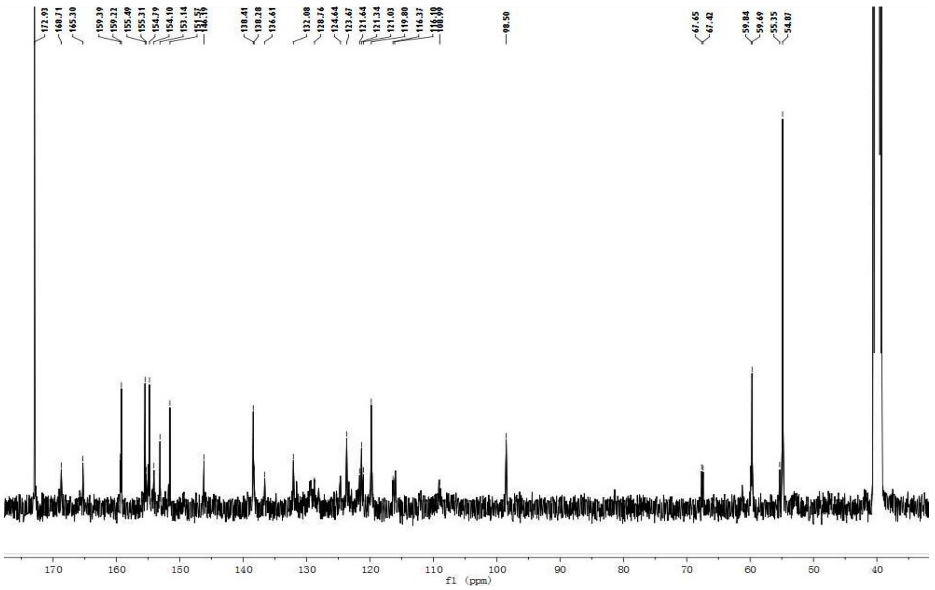

Fig. S4. ^{13}C NMR spectrum of the ligand **TR-NO** (100 MHz, DMSO-d_6).

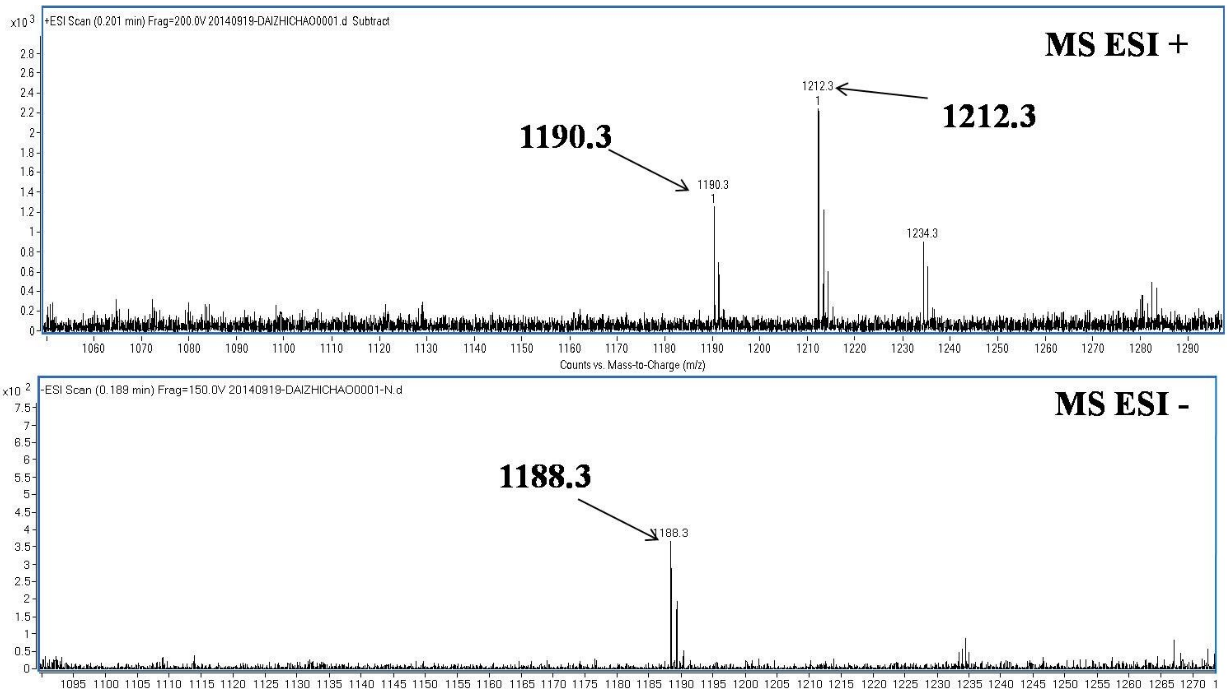

Fig. S5. Mass spectra of the product of **TRP-NO** reacted with NO in 0.05 M PBS buffer of pH 7.4.

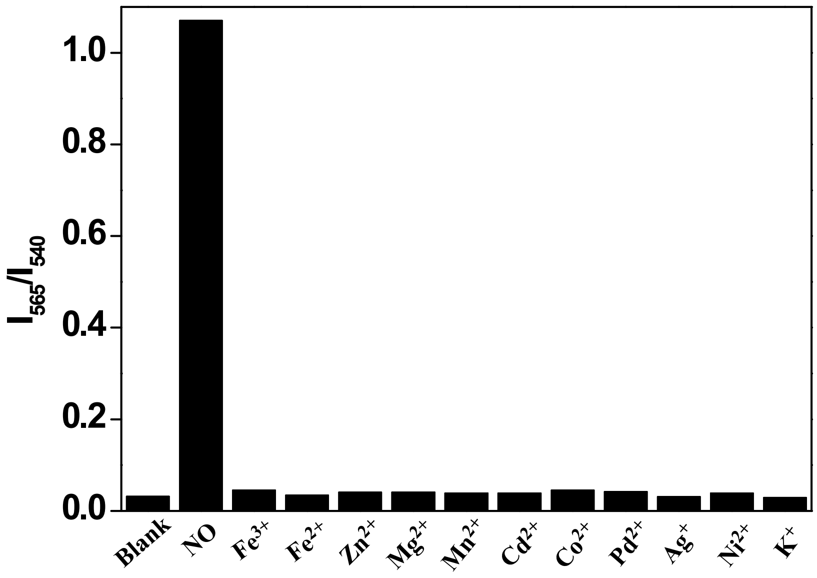

Fig. S6. Effects of some metal ions (100 μM) on the I_{565}/I_{540} ratio of **TRP-NO** (15 μM) in 0.05 M PBS buffer of pH 7.4 for 50 min.

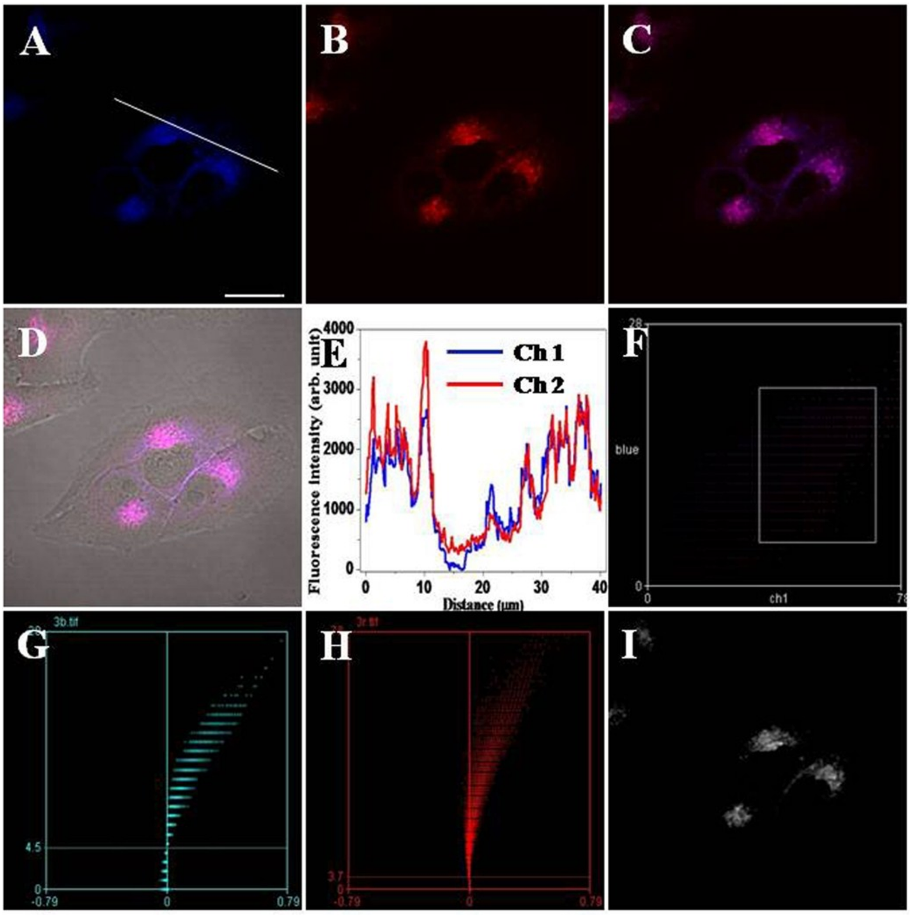

Fig. S7. Intracellular co-localization analysis of **TRP-NO** and LysoTracker Blue in HepG2 cells. **A-C:** Images of HepG2 cells that were incubated with **TRP-NO**, treated with NOC-13 and further stained with LysoTracker Blue (**A**: channel 1, λ_{ex} = 405 nm, λ_{em} = 415 - 475 nm; **B**: channel 2, λ_{ex} = 559 nm, λ_{em} = 590-640 nm; **C**: overlay of **A** and **B**). **D:** Merged images of **C** with its DIC. **E:** Luminescence intensity profiles of the interest linear region across HepG2 cells in **A**. **F:** Intensity correlation plot of HepG2 cells that were co-localized with **TRP-NO** and LysoTracker Blue. **G:** ICA plots of LysoTracker Blue-stained cells. **H:** ICA plots of **TRP-NO**-stained cells. (i) PDM image of the cells with positive values in the pixels. Scale bar, 10 μm.

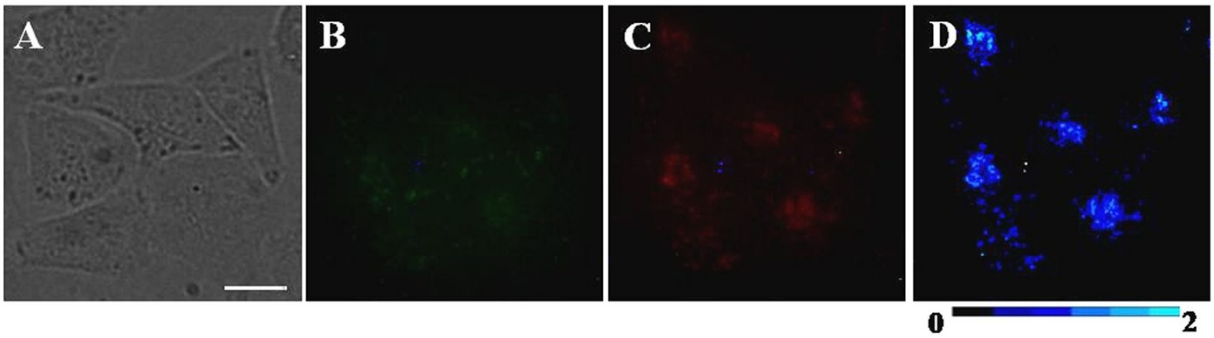

Fig. S8. Time-gated luminescence images of the **TRP-NO**-loaded RAW 264.7 macrophage cells after stimulation with LPS. **A:** bright-field image; **B:** Tb^{3+} luminescence image; **C:** rhodamine luminescence image; **D:** ratiometric (ratio = I_{red}/I_{green}) luminescence image. Scale bar: 10 μm.

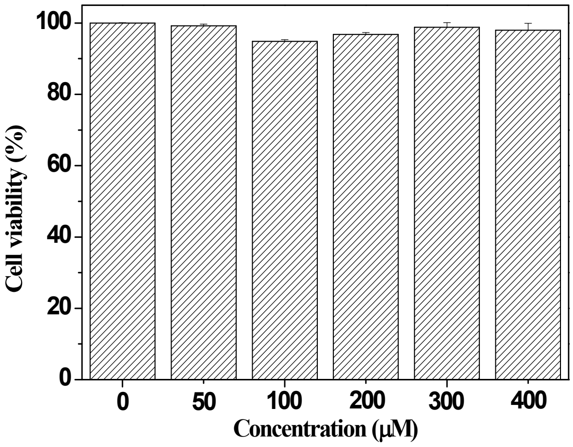

Fig. S9. Viabilities of HepG2 cells after incubation with different concentrations of **TRP-NO** for 4 h.

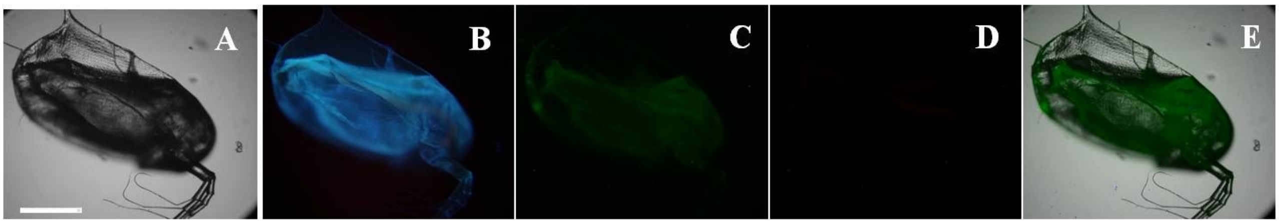

Fig. 10. Bright-field (A), steady-state (B) and time-gated (C for Tb^{3+} luminescence, D for rhodamine luminescence) luminescence images of the **TRP-NO**-loaded *D. magna* before incubation with NO. Image E is the merged image of A and C. Scale bar: 200 μm.
